# Critical Relations of Crowns in Critical Times of Coronavirus Depression

**DOI:** 10.1007/s11083-021-09571-6

**Published:** 2021-07-07

**Authors:** Ádám Kunos, Miklós Maróti, László Zádori

**Affiliations:** 1 Bolyai Institute, University of Szeged, Szeged, Hungary; 2 Alfréd Rényi Institute of Mathematics, Budapest, Hungary

**Keywords:** Poset, Crown, Clone, Finitely generated, Obstruction, Critical relation, Subpower membership problem

## Abstract

The critical relations are the building blocks of the relational clone of a relational structure with respect to the relational operations intersection and direct product. In this paper we describe the critical relations of crowns. As a consequence, we obtain that the subpower membership problem for any crown is polynomial-time solvable.

## 1 Introduction

The relational structures in this note are assumed to be always finite. A *crown* is a height 1 poset whose comparabilty graph is a cycle, see Fig. 1.

Let $\mathbf{m}$ denote $m$-element antichain and $+$ the linear sum of posets. Let

$$\overline{\mathscr{P}} = \mathbf{1}\text{+}\mathbf{2}\text{+}\mathscr{P}\text{+}\mathbf{2}\text{+}\mathbf{1}$$

where $\mathscr{P}$ is a poset, and let $\mathscr{T} = \overline{\mathbf{2}}$. In his seminal paper [10] Tardos proved that the clone of the eight element poset $\mathscr{T}$ is not finitely generated. Following the terminology in [8], we call the posets of the form $\overline{\mathscr{C}}$ where $\mathscr{C}$ is a crown *locked crowns*, see Fig. 2.

The research of authors was supported by the grants TUDFO/47138-1/2019-ITM and NKFIH-1279-2/2020 of the Ministry for Innovation and Technology, Hungary, the EU-funded Hungarian grant EFOP-3.6.2-16-2017-00015, and the NKFIH grants K115518 and K128042. The third author was also supported by the Alfréd Rényi Institute of Mathematics, ELKH, Hungary.

✉ Ádám Kunos
akunos@math.u-szeged.hu

Miklós Maróti
mmaroti@math.u-szeged.hu

László Zádori
zadori@math.u-szeged.hu

[1] Bolyai Institute, University of Szeged, Szeged, Hungary

[2] Alfréd Rényi Institute of Mathematics, Budapest, Hungary

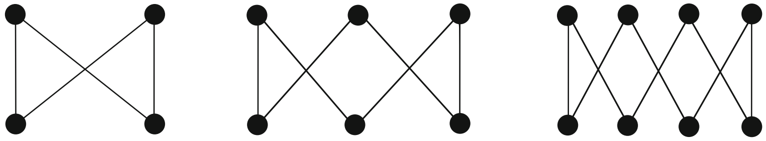

**Fig. 1** Some crowns of small cardinality

It is an open question related to Tardos's result whether the clone of every locked crown is non-finitely generated. The positive answer to this question would be somewhat surprising at the first sight, since Demetrovics and Rónyai have proved in [3] that the clone of monotone operations of any crown is finitely generated. However, from results of the third author in [13] it follows that the clone of $\overline{\mathscr{C}}$ is non-finitely generated if $\mathscr{C}$ is the four element crown. The proof of this fact in [13] is reduced to Tardos's original proof for the poset $\mathscr{T}$. Unfortunately, we are not able to use Tardos's method to decide whether the clone of $\overline{\mathscr{C}}$ is finitely generated or not if $\mathscr{C}$ has at least six elements. One of the reasons why Tardos's proof works is that the extendibility of a partial map from a finite power of $\mathscr{T}$ to $\mathscr{T}$ can be easily decided by checking some minimal non-extendible configurations called obstructions. The obstructions have a very simple structure for $\mathscr{T}$, but they are unwieldy for $\overline{\mathscr{C}}$ where $\mathscr{C}$ is a crown. A possible cause for the latter is that the decision problem **Ext**($\mathscr{C}$) introduced in the next paragraph is **NP**-complete for any crown $\mathscr{C}$. To circumvent this problem we plan to use the critical relations of $\overline{\mathscr{C}}$ instead of its obstructions to verify the extendibility of a partial map from a finite power of $\overline{\mathscr{C}}$ to $\overline{\mathscr{C}}$. The obstructions of $\overline{\mathscr{C}}$ are easily obtained from the obstructions of $\mathscr{C}$, the latter ones have no tangible description, though, as we have mentioned. At present, we do not know an easy way to get the critical relations of $\overline{\mathscr{C}}$ from the critical relations of $\mathscr{C}$, but in this note we at least give a simple description of the critical relations of any crown $\mathscr{C}$. We hope that this leads to a description of the critical relations of $\overline{\mathscr{C}}$ and, eventually, to a decision whether the clone of $\overline{\mathscr{C}}$ is finitely generated.

In [7], Larose and the third author introduced a decision problem called the *extendibility problem* **Ext**($\mathscr{P}$) *for a finite poset* $\mathscr{P}$: given a finite poset $\mathscr{Q}$ and a partial map $f$ from $\mathscr{Q}$ to $\mathscr{P}$, decide whether $f$ extends to a monotone total map from $\mathscr{Q}$ to $\mathscr{P}$. We define the *graph of* $f$ to be the set $\{(h, f(h)) : h \in \mathrm{Dom}(f)\}$. So an input of **Ext**($\mathscr{P}$) is the pair formed by a poset $\mathscr{Q}$ and the graph of $f$. It is easy to see that **Ext**($\mathscr{P}$) is in the complexity class **NP**. Moreover, Pratt and Tiuryn proved in [9] that **Sat**($\mathscr{C}$), a polynomial-time equivalent of **Ext**($\mathscr{C}$), is **NP**-complete if $\mathscr{C}$ is a crown. Thus **Ext**($\mathscr{C}$) is **NP**-complete if $\mathscr{C}$ is a crown. In [7], this result was extended for other posets characterized by an algebraic condition.

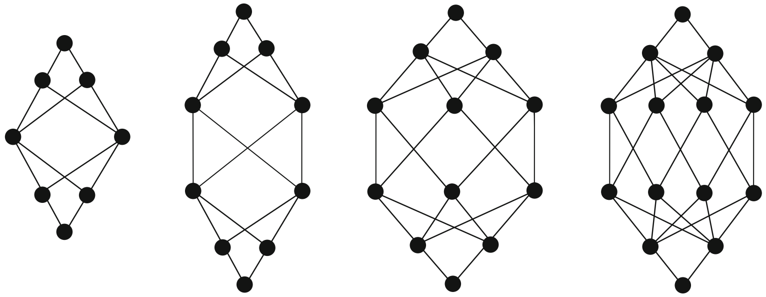

**Fig. 2** Poset $\mathscr{T}$ and some locked crowns of small cardinality

When studying finite generability for the clone of a finite poset $\mathscr{P}$, often an other kind of extendibility question occurs: given a power $\mathscr{P}^n$ of $\mathscr{P}$ and a partial map $f$ from $\mathscr{P}^n$ to $\mathscr{P}$, decide whether $f$ extends to a monotone total map from $\mathscr{P}^n$ to $\mathscr{P}$. We call this problem the *restricted extendibility problem for* $\mathscr{P}$ and denote it by **RExt**($\mathscr{P}$). Thus, an input of this problem is the pair formed by the poset $\mathscr{P}^n$ and the graph of $f$. Clearly, **RExt**($\mathscr{P}$) is a polynomial-time reducible to **Ext**($\mathscr{P}$), so it is in **NP**.

An *order-primal algebra related to a poset* $\mathscr{P}$ is an algebra whose base set equals that of $\mathscr{P}$ and whose term operations coincide with the monotone operations of $\mathscr{P}$.

We consider another problem, a variant of the restricted extendibility problem. The new problem is called the *subpower membership problem* **SMP**($\mathscr{P}$): given a partial map $f$ from $\mathscr{P}^n$ to $\mathscr{P}$, decide whether $f$ extends to a monotone total map from $\mathscr{P}^n$ to $\mathscr{P}$. Thus, an input of this problem is just the graph of $f$. Notice that the subpower membership problem is thriftier with the sizes of the inputs than the restricted extendibility problem. It is evident that **RExt**($\mathscr{P}$) is polynomial-time reducible to **SMP**($\mathscr{P}$).

Let us suppose now that an input of **SMP**($\mathscr{P}$) is given by the list of pairs $(h_i, f(h_i))$, $1 \leq i \leq k$, where $h_i$ runs through the domain of $f$ in $P^n$. Alternatively, we may conceive this list as an $(n+1)$-element set of $k$-tuples where the $j$-th $k$-tuple, $j \leq n$, is the tuple determined by the $j$-th coordinates of the $h_i$ and the $(n+1)$-st $k$-tuple is $(f(h_1), \ldots, f(h_k))$, and then the question of **SMP**($\mathscr{P}$) becomes whether the subalgebra generated by the first $n$ tuples in the $k$-th power of an order-primal algebra related to $\mathscr{P}$ contains the $(n+1)$-st $k$-tuple. The study of these types of problems was first suggested by Willard, see [11], in an algebraic and more general setting. Since then, the research in this area has become a burgeoning branch of mathematics, see [2] for further details and references on the subpower membership problem.

In the proof of the characterization of critical relations of crowns, one of the main tools is a result of Demetrovics and Rónyai in [3] that states that every monotone surjective operation of a crown is essentially unary. As a consequence of our result, we also prove that **SMP**($\mathscr{C}$), and hence **RExt**($\mathscr{C}$), is solvable in polynomial time if $\mathscr{C}$ is a crown. This gives sharp contrast between the complexities of the two decision problems **Ext**($\mathscr{P}$) and **RExt**($\mathscr{P}$). We do not know the answer to the question if there is a finite poset $\mathscr{P}$ for which **RExt**($\mathscr{P}$) is **NP**-complete.

## 2 Critical Relations and Obstructions of Relational Structures

In this section we introduce some basic definitions and prove some general claims on critical relations and obstructions of finite structures. These statements will be applied in the later sections when studying the critical relations of crowns.

Let $\mathscr{R}$ be a relational structure. We denote the $n$-th *power* of $\mathscr{R}$ by $\mathscr{R}^n$. The relational structure $\mathscr{R}^n$ has the same type as $\mathscr{R}$. Its base set is $R^n$ where $R$ is the base set of $\mathscr{R}$. Each of its $k$-ary relations $\rho_{R^n}$ is obtained from a $k$-ary relation $\rho_{\mathscr{R}}$ of $\mathscr{R}$ by

$$(r_1, \ldots, r_k) \in \rho_{R^n}$$

if and only if all of the $k$-tuples defined coordinatewise from the $n$-tuples $r_1, \ldots, r_k$ are in $\rho_{\mathscr{R}}$.

Let $\mathscr{S}$ be a relational structure of the same type as $\mathscr{R}$. We say that a map $f : S \to R$ is a *homomorphism* from $\mathscr{S}$ to $\mathscr{R}$ if $f$ *preserves the relations*, that is, for any $k$-ary relation $\rho_{\mathscr{S}}$ of $\mathscr{S}$ and any $k$-tuple $(s_1, \ldots, s_k) \in \rho_{\mathscr{S}}$, we also have $(f(s_1), \ldots, f(s_k)) \in \rho_{\mathscr{R}}$. An *isomorphism* is a bijective homomorphism whose inverse is also a homomorphism. A

homomorphism from $\mathscr{R}^n$ to $\mathscr{R}$ is called a *polymorphism* of $\mathscr{R}$. The set of polymorphisms of $\mathscr{R}$ is called the *clone* of $\mathscr{R}$ and is denoted by $\mathrm{Clo}(\mathscr{R})$. The set of $n$-ary polymorphisms in $\mathrm{Clo}(\mathscr{R})$ is denoted by $\mathrm{Clo}^n(\mathscr{R})$. A unary polymorphism of a relational structure is called an *endomorphism.* An *automorphism* is a bijective endomorphism.

Let $F$ be a set of operations on a set $R$. A $k$-ary relation $\rho$ is called an *invariant relation* with respect to $F$ if all the operations of $F$ are polymorphisms of the relational structure $(R; \rho)$. In this case, we say that the operations in $F$ *preserve* $\rho$. The set of all invariant relations with respect to $F$ is denoted by $\mathrm{Inv}(F)$. The set of $k$-ary relations in $\mathrm{Inv}(F)$ is denoted by $\mathrm{Inv}^k(F)$. We remark that $\mathrm{Inv}^k(F)$ is a lattice with respect to containment. For a relational structure $\mathscr{R}$, $\mathrm{Inv}(\mathrm{Clo}(\mathscr{R}))$ is called the *relational clone of* $\mathscr{R}$.

In this paper, a *direct product* of a $k$-ary relation $\alpha$ and an $l$-ary relation $\beta$ is a relation obtained by permutation of coordinates from the relation

$$\{(a_1, \ldots, a_k, b_1, \ldots, b_l)|\ (a_1, \ldots, a_k) \in \alpha,\ (b_1, \ldots, b_l) \in \beta\}.$$

We use the notation $\alpha \times \beta$ to denote any of the direct products of $\alpha$ and $\beta$, from the context it will always be clear which of the direct products we think of.

A relation $\rho \in \mathrm{Inv}^k(F)$ is *meet-irreducible* if it is not a proper meet of any two invariant relations of $\mathrm{Inv}^k(F)$. Following the terminology in [6], we call a relation $\rho \in \mathrm{Inv}^k(F)$ a *critical relation* with respect to $F$ if it is meet-irreducible in the lattice $\mathrm{Inv}^k(F)$ and is not a direct product of two relations with smaller arities. Let $\mathrm{Crit}(\mathscr{R})$ denote the set of the critical relations with respect to $\mathrm{Clo}(\mathscr{R})$. We call the elements of $\mathrm{Crit}(\mathscr{R})$ the *critical relations of* $\mathscr{R}$. The definition of $\mathrm{Crit}^{\mathrm{k}}(\mathscr{R})$ is analogous.

**Lemma 1** *Let $\mathscr{R}$ be a finite relational structure and $\rho \in \mathrm{Inv}^k(\mathrm{Clo}(\mathscr{R}))$ a meet-irreducible relation. Then $\rho$ is a critical relation or a direct product of a critical relation and a finite power of the base set of $\mathscr{R}$.*

*Proof* Suppose that $\rho$ is a meet-irreducible but not a critical relation. Then $\rho$ is of the form $\alpha \times \beta$ for suitable relations $\alpha,\ \beta \in \mathrm{Inv}(\mathrm{Clo}(\mathscr{R}))$. Let $\alpha$ be an $l$-ary and $\beta$ an $m$-ary relation where $l+m = k$. Then $\rho = (R^l \times \beta) \cap (\alpha \times R^m)$. By meet irreducibility, either $\rho = R^l \times \beta$ or $\rho = \alpha \times R^m$. Without loss of generality, we assume that $\rho = \alpha \times R^m$. Now, $\alpha$ must be meet-irreducible, for otherwise $\rho$ would not be meet-irreducible. Thus, $\alpha$ is a meet-irreducible relation with a smaller arity than $\rho$. Hence, an induction on the arity of $\rho$ yields the claim. $\square$

Let $0_R$ denote the equality relation of a set $R$. We say that $\rho \in \mathrm{Inv}^{\mathrm{k}}(\mathrm{Clo}(\mathscr{R}))$ *has a repetition of coordinates* if there exist two different coordinates such that by projecting $\rho$ to these coordinates, the resulting binary relation is contained by $0_R$.

**Lemma 2** *Let $\mathscr{R}$ be a finite relational structure and $\rho \in \mathrm{Inv}^k(\mathrm{Clo}(\mathscr{R}))$ a meet-irreducible relation. Then $\rho$ has no repetition of coordinates or it is of the form $R^{k-2} \times 0_R$. So every critical relation of arity at least three has no repetition of coordinates.*

*Proof* If $|R| = 1$, then the claim is trivial. Suppose that $|R| \geq 2$ and the last two coordinates of the tuples in $\rho$ are the same. Let $\alpha$ be the projection of $\rho$ to the first $k-1$ coordinates. Then $\rho = (\alpha \times R) \cap (R^{k-2} \times 0_R)$. Since $\rho$ is meet irreducible and $\rho \neq \alpha \times R$, we have that $\rho = R^{k-2} \times 0_R$. $\square$

Later, we shall work in the class of posets. It is clear that this class is closed under finite power. Now, we are going to prove two theorems for classes $\mathscr{K}$ of general relational structures such that $\mathscr{K}$ is closed under finite power. These theorems play a fundamental role in the next section. First, we require a relativized version of a basic representation theorem for the invariant relations of a relational structure, cf. [4].

**Theorem 3** *Let $\mathscr{K}$ be a class of models in a fixed language of relational structures such that $\mathscr{K}$ is closed under finite power. Let $\mathscr{R}$ be a finite relational structure in $\mathscr{K}$ and $\rho$ a non-empty k-ary relation on R. Then $\rho \in \mathrm{Inv}^k(\mathrm{Clo}(\mathscr{R}))$ if and only if*

$$\rho = \{(h(s_1), \ldots, h(s_k)) \mid h : \mathscr{S} \to \mathscr{R} \quad \textit{is a homomorphism}\} \tag{1}$$

*for some finite relational structure $\mathscr{S} \in \mathscr{K}$ and elements $s_1, \ldots, s_k \in S$.*

*Proof* The sufficiency part is clear as a composition of homomorphisms is a homomorphism itself. We prove the necessity part of the theorem. So let $\rho \in \mathrm{Inv}^k(\mathrm{Clo}(\mathscr{R}))$ and let $n = |\rho|$. The elements of $\rho$ as columns constitute a $k \times n$ matrix, let $s_1, \ldots, s_k \in R^n$ be the rows of this matrix in the natural order and let $\mathscr{S} = \mathscr{R}^n$. We note that $\mathscr{S} \in \mathscr{K}$, since $\mathscr{K}$ is closed under finite power. To see that Eq. 1 holds observe that each homomorphism from $\mathscr{S}$ to $\mathscr{R}$ is an $n$-ary operation in $\mathrm{Clo}(\mathscr{R})$ that preserves $\rho$, hence $\rho$ contains the right hand side of Eq. 1. By restricting the $n$-ary projections—that are homomorphisms from $\mathscr{S}$ to $\mathscr{R}$— onto $s_1, \ldots, s_k$, one sees that the converse containment also holds. □

If $\rho$, $\mathscr{S}$ and $s_1, \ldots s_k \in S$ are as the ones in the statement of the preceding theorem, we say that $\mathscr{S}$ *defines $\rho$ on $s_1, \ldots, s_k$.*

For sets $H$ and $P$, the partial maps $f : H \rightharpoonup P$ are called the *colorings of $H$ with $P$.* By a *coloring of a relational structure*, we naturally mean a coloring of its base set. We call the pair $(\mathscr{S}, f)$ an *$\mathscr{R}$-colored structure* if $f$ is a partial map from $\mathscr{S}$ to $\mathscr{R}$. An $\mathscr{R}$-colored structure $(\mathscr{S}, f)$ is called *extendible* if there exists a homomorphism $g : \mathscr{S} \to \mathscr{R}$ which extends $f$. Let $\mathscr{R}$ and $\mathscr{S}$ be relational structures of the same type with base sets $R$ and $S$, respectively. We say that $\mathscr{R}$ *contains* $\mathscr{S}$ if $R \supseteq S$ and each relation of $\mathscr{R}$ contains the corresponding relation of $\mathscr{S}$.

Let $\mathscr{K}$ be a class of models in a fixed language of relational structures such that $\mathscr{K}$ is closed under finite power and $\mathscr{R}$ a finite relational structure in $\mathscr{K}$. An $\mathscr{R}$-colored structure $(\mathscr{S}, f)$ is called an *$\mathscr{R}$-obstruction* if $\mathscr{S}$ is a finite relational structure in $\mathscr{K}$, $(\mathscr{S}, f)$ is not extendible, but for all $\mathscr{S}' \in \mathscr{K}$ properly contained in $\mathscr{S}$, $(\mathscr{S}', f|_{S'})$ is extendible.

The following theorem roughly says that to every critical relation we can assign an obstruction whose base relational structure defines it on the colored elements. A loose interpretation of this is that there are more obstructions than critical relations.

**Theorem 4** *Let $\mathscr{K}$ be a class of models in a fixed finite language of relational structures such that $\mathscr{K}$ is closed under finite power and $\mathscr{R}$ a finite relational structure in $\mathscr{K}$. If $\rho \in \mathrm{Crit}^{\mathrm{k}}(\mathscr{R})$ and $\rho \neq \emptyset, 0_R$, then there exist a finite relational structure $\mathscr{S} \in \mathscr{K}$ and an $\mathscr{R}$-obstruction $(\mathscr{S}, f)$ with $\mathrm{Dom}(f) = \{s_1, \ldots, s_k\}$ such that $\mathscr{S}$ defines $\rho$ on $s_1, \ldots, s_k$.*

*Proof* We apply Theorem 3 as follows. We choose a finite $\mathscr{S} \in \mathscr{K}$ such that $\mathscr{S}$ defines $\rho$ on $s_1, \ldots, s_k \in S$ and $\mathscr{S}$ is minimal with respect to containment among the finite structures in $\mathscr{K}$ that define $\rho$. For every $\mathscr{S}' \in \mathscr{K}$ where $\mathscr{S}' \subset \mathscr{S}$, let $\mathscr{S}''$ denote the structure we get from $\mathscr{S}'$ by changing $S'$ to $S' \cup \{s_1, \ldots, s_k\}$ and retaining the relations of $\mathscr{S}'$ on $S' \cup \{s_1, \ldots, s_k\}$.

Let

$$\rho_{\mathscr{S}'} = \{(h(s_1), \ldots, h(s_k)) \mid h : \mathscr{S}'' \to \mathscr{R} \quad \text{is a homomorphism}\}.$$

It is obvious that $\rho_{S'}$ is an invariant relation of $\mathscr{R}$ and that

$$\rho \subseteq \rho_{\mathscr{S}'}. \tag{2}$$

We claim that this inclusion is proper. If $\{s_1, \ldots, s_k\} \subseteq S'$, then the minimality of $\mathscr{S}$ implies the proper inclusion. If $\{s_1, \ldots, s_k\} \nsubseteq S'$, then $\rho_{\mathscr{S}'}$ is obviously a direct product, so $\rho \neq \rho_{\mathscr{S}'}$, which together with Eq. 2 yields that $\rho \subset \rho_{\mathscr{S}'}$ indeed.

Let $\rho'$ be the unique cover of $\rho$. Then, by using the proper inclusions we have just proved, we obtain that

$$\rho \subset \rho' \subseteq \bigcap_{\mathscr{S}' \subset \mathscr{S},\ \mathscr{S}' \in \mathscr{K}} \rho_{\mathscr{S}'}.$$

Let $r = (r_1, \ldots, r_k) \in \rho' \setminus \rho$. We define the partial map $f$ on $S$ by $f : s_i \mapsto r_i$, $1 \leq i \leq k$. We have to argue that $f$ indeed is a map from its domain. By Lemma 2, it suffices to consider the case when $k = 2$ and $s_1 = s_2$. Since $\rho \neq 0_R$ and $\mathscr{S}$ defines $\rho$ on $s_1 = s_2$, $\rho$ is a proper subset of $0_R$. Let $(r, r) \notin \rho$. Then the invariant relation generated by $\rho \cup \{(r, r)\}$ contains $\rho'$ and, on the other hand, is a subset of the equality, hence $r_1 = r_2$. Thus, $f$ is a partial map.

We claim that $(\mathscr{S}, f)$ is an $\mathscr{R}$-obstruction. From $r \notin \rho$, we see that $(\mathscr{S}, f)$ is not extendible. Moreover, for any $\mathscr{S}' \subset \mathscr{S}$, $\mathscr{S}' \in \mathscr{K}$, we have $r \in \rho_{\mathscr{S}'}$ and hence $(\mathscr{S}', f\big|_{S'})$ is extendible. □

The next theorem highlights the significance of critical relations. Let $(\mathscr{R}^n, f)$ be a finite $\mathscr{R}$-colored structure and $\rho$ a $k$-ary invariant relation of $\mathscr{R}$. We say that $f$ *preserves* $\rho$ if

$$\text{for all } r_1, \ldots, r_k \in \mathrm{Dom}(f),\ (f(r_1), \ldots, f(r_k)) \in \rho \text{ whenever } (r_1, \ldots, r_k) \in \rho. \tag{3}$$

**Theorem 5** *Let $(\mathscr{R}^n, f)$ be a finite $\mathscr{R}$-colored structure. Then $f$ is extendible if and only if for all $\rho \in \mathrm{Crit}^k(\mathscr{R})$, $f$ preserves $\rho$.*

*Proof* The "only if" direction is obvious as critical relations are invariant. For the "if" part, first, let us observe that property (3) is inherited for direct product and intersection of relations. Moreover, all invariant relations are obtained from critical relations with direct product and intersection. So $f$ preserves all invariant relations instead of critical relations. This implies that if $\mathrm{Dom}(f) = \{a_1, \ldots, a_m\}$ and $(a_1, \ldots, a_m)$ is in an invariant relation $\alpha \subseteq R^m$, then $(f(a_1), \ldots, f(a_m)) \in \alpha$ as well. For the specific

$$\alpha = \left\{(h(a_1), \ldots h(a_m)) \middle| \ h : \mathscr{R}^n \to \mathscr{R} \quad \text{is a homomorphism}\right\}, \tag{4}$$

it is clear (taking the projections) that $(a_1, \ldots, a_m)$ is in $\alpha$, hence from our previous observation $(f(a_1), \ldots, f(a_m)) \in \alpha$ as well. Then, by the definition in Eq. 4, there is a homomorphism extending $f$. □

Let $\mathscr{K}$ be a class of models in a fixed finite language of relational structures such that $\mathscr{K}$ is closed under finite power and $\mathscr{R}$ a finite relational structure in $\mathscr{K}$. Notice that the set of $\mathscr{R}$-obstructions depends on the class $\mathscr{K}$, while the set of critical relations only depends on $\mathscr{R}$. By Theorem 5, it suffices to know the critical relations of $\mathscr{R}$ for deciding the extendibility of an $\mathscr{R}$-colored structure $(\mathscr{R}^k, f)$. On the other hand, the extendibility of $(\mathscr{R}^k, f)$ can be checked by the use of obstructions as well: $(\mathscr{R}^k, f)$ is extendible if and only if it contains no $\mathscr{R}$-obstructions. By Theorem 4, in a way, there are less critical relations than obstructions, and sometimes it is much easier to describe the critical relations in $\mathrm{Crit}(\mathscr{R})$ than the $\mathscr{R}$-obstructions. So the extendibility of $(\mathscr{R}^k, f)$ might turn out to be easier to decide by the use of critical relations.

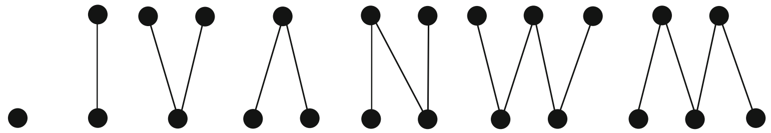

**Fig. 3** Fences of small cardinality

In the next two sections, we supply a description of the critical relations of the crowns. This is of significance since no tangible description is known for the obstructions of crowns within the class of posets. This is how we proceed: first we describe the binary critical relations of crowns and then we settle the general case. As an application, we prove that there is a polynomial-time algorithm that solves the subpower membership problem for any crown. A *fence* is a one element poset or a length 1 poset whose comparability graph is a path, see Fig. 3. Let $\mathscr{F}_k$ denote the $(k+1)$-element fence (when $k$ is even there are two such posets dual to each other, then $\mathscr{F}_k$ denotes any of them), and let $\mathscr{C}_k$ denote the $2k$-element crown.

## 3 The Binary Critical Relations of $\mathscr{C}_k$

From now on, we work in the class of posets. All of the definitions and statements introduced in the preceding section are valid for posets as they constitute a class closed under finite power. In this section, we use Theorem 4 to describe the binary critical relations of $\mathscr{C}_k$. We remark that the description of the binary critical relations of $\mathscr{C}_k$ can also be obtained without Theorem 4, by using elementary facts about unary monotone operations of $\mathscr{C}_k$ and determining the smallest invariant relation containing a pair $(a, b) \in \mathscr{C}_k^2$.
Our starting point to obtain such a description is an earlier result in [12]. A ternary operation $m$ is a *majority operation* if it satisfies the identities

$$m(x, y, y) = m(y, x, y) = m(y, y, x) = y.$$

**Fig. 4** The lattice of binary invariant relations of $\mathscr{C}_k$

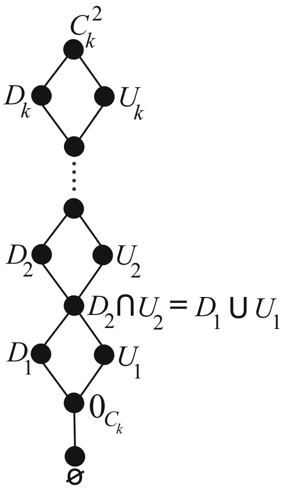

It is a well known fact that the fences admit a majority operation, so by Proposition 3.1 in [12] we have the following.

**Lemma 6** *The $\mathscr{F}_k$-obstructions are the non-extendible $\mathscr{F}_k$-colored fences that are colored at the two endpoints.*

**Theorem 7** *The $\mathscr{C}_k$-obstructions with two colored elements are the non-extendible $\mathscr{C}_k$-colored fences that are colored at the two endpoints.*

*Proof* Let $(\mathscr{H}, f)$ be a $\mathscr{C}_k$-obstruction with two colored elements colored by $a$ and $b$. Clearly, $a$ and $b$ are different. Let $\mathscr{F}$ be a shortest path connecting $a$ and $b$ in $C_k$. Then $\mathscr{F}$ is a fence and $(\mathscr{H}, f)$ is a nonextendible $\mathscr{F}$-colored poset. So it contains an $\mathscr{F}$-obstruction $(\mathscr{H}', f)$ such that $\mathscr{H}'$ is a fence whose endpoints are colored by $a$ and $b$. By the preceding lemma, $\mathscr{H}'$ has length shorter than $\mathscr{F}$, or $\mathscr{H}'$ is the dual of $\mathscr{F}$. In both cases $(\mathscr{H}', f)$ is a non-extendible $\mathscr{C}_k$-colored poset, since $\mathscr{F}$ is a shortest path connecting $a$ and $b$ in $\mathscr{C}_k$. By the minimality of $(\mathscr{H}, f)$, $(\mathscr{H}, f) = (\mathscr{H}', f)$ and we are done. □

In a connected poset $\mathscr{Q}$, for any two elements $a$ and $b$ we define the updistance of $a$ from $b$ to be the least positive integer $n$ such that there exists a sequence of elements $a = a_0 \leq a_1 \geq a_2 \leq \dots a_n = b$ in $\mathscr{Q}$. The downdistance of $a$ from $b$ is defined dually. We let $\uparrow (a, b)$ and $\downarrow (a, b)$ denote the up and downdistance from $a$ to $b$, respectively. We define some binary relations in the relational clone of $\mathscr{C}_k$: let

$$U_l = \{(a, b) : \uparrow (a, b) \leq l\} \text{ and } D_l = \{(a, b) : \downarrow (a, b) \leq l\}$$

where $l \leq k$.

Notice that the unary invariant relations of a finite poset $\mathscr{P}$ are $\emptyset$ and $P$, hence the only unary critical relation is $\emptyset$. Therefore, the only direct product relations in $\mathrm{Inv}^2(\mathrm{Clo}(\mathscr{P}))$ are $\emptyset$ and $\mathscr{P}^2$.

**Theorem 8** *The binary critical relations of $\mathscr{C}_k$ are $U_l$ and $D_l$ where $l \leq k$.*

*Proof* Notice that if a poset is not an antichain, then its equality relation is not critical since it is the intersection of $\leq$ and $\leq^{-1}$. So by Theorem 4 and Theorem 7, every binary critical relation of $\mathscr{C}_k$ is of the form $U_l$ or $D_l$ for some $l \leq k$. By the remark preceding the theorem, the only nonempty direct product relation in $\mathrm{Inv}^2(\mathrm{Clo}(\mathscr{C}_k))$ is $C_k^2$ and all of the $U_l$ and $D_l$ are smaller than $C_k^2$.

To conclude the proof, it suffices to verify that the $U_l$ and $D_l$ are meet-irreducible. Observe that

$$U_l,\ D_l\ \leq\ U_{l+1},\ D_{l+1}$$

for all $l \leq k-1$, and $U_l$ and $D_l$ are incomparable for all $l \leq k$. Moreover

$$U_{l+1} \cap D_{l+1} = U_l \cup D_l.$$

Notice that we have just described the lattice of binary invariant relations of $\mathscr{C}_k$, see Fig. 4. Thus, $U_l$ and $D_l$ are meet-irreducible indeed for all $l \leq k$. □

## 4 The $n$-ary Critical Relations of $\mathscr{C}_k$ for $n \geq 3$

In this section we complete the characterization of the critical relations of crowns. Clearly, the automorphism group of $\mathscr{C}_k$ is isomorphic to the $2k$-element dihedral group if $k \geq 3$, and is isomorphic to the square of the two element group if $k = 2$. Observe that the range of any non-onto polymorphism of $\mathscr{C}_k$ is an at most $(k+1)$-element fence due to the fact that the largest down (up) distance is $k$ in $\mathscr{C}_k$, and this property and connectedness are inherited for the powers of $\mathscr{C}_k$ and their monotone images. We call an endomorphism $f$ of a poset a *retraction* if $f^2 = f$. A *retract* of a poset $P$ is a poset that is isomorphic to the image of a retraction of $P$. By coincidence, every at most $(k+1)$-element fence is a retract of $\mathscr{C}_k$.

The *Słupecki relation* $S_k$ on a $k$-element set where $k \geq 3$ is the set of all $k$-tuples that have at most $k-1$ pairwise different components. It is well known that a surjective operation $f$ preserves $S_k$ if and only if $f$ is essentially unary. So the clone of operations that preserve $S_k$ consists of the unary operations and the non-surjective operations of the $k$-element set when $k \geq 3$. This clone is called the *Słupecki clone*.

We require a result of Demetrovics and Rónyai which states that the clone of every crown is contained in the Słupecki clone, see Theorem 3.3 in [3]. An equivalent formulation of their theorem asserts that the surjective monotone operations of crowns are essentially unary. In the proof of Theorem 3.3 in [3], Demetrovics and Rónyai exhibited a poset that defines the Słupecki relation on suitable sequence of elements, proving in this way that the relational clone of $\mathscr{C}_k$ contains the Słupecki relation. For completeness, here we give another proof of their theorem by the use of Jablonskiy's lemma in [5].

**Lemma 9** (Jablonskiy, [5]) *For $k \geq 3$, let $A$ be an $k$-element set and $f : A^n \to A$ an onto operation that depends on at least two of its variables. Then there exist $(k-1)$-element subsets $A_1, \dots, A_n$ of $A$ such that $f(A_1 \times \cdots \times A_n) = A$.*

**Theorem 10** (Demetrovics and Rónyai, [3]) *In the clone of $\mathscr{C}_k$, the only $n$-ary surjective operations are the ones of the form $\alpha(\pi_i)$, where $\alpha$ is an automorphism of $\mathscr{C}_k$ and $\pi_i$ is the projection to the $i$-th coordinate where $1 \leq i \leq n$.*

*Proof* Suppose that $f$ is an $n$-ary monotone onto operation of $\mathscr{C}_k$ that depends on at least two of its variables. We set $A = C_k$ and apply Jablonskiy's lemma. By leaving out an element from $\mathscr{C}_k$ we obtain a $(2k-1)$-element fence. So the subposets $\mathscr{A}_1, \dots, \mathscr{A}_n$ induced by $A_1, \dots, A_n$ in $\mathscr{C}_k$ are $(2k-1)$-element fences. Notice that there is an element (the middle element) in these fences such that both the updistance and the downdistance from this special element to each element is at most $k$. This property is inherited for the product $\mathscr{A}_1 \times \cdots \times \mathscr{A}_n$ and, since $f$ is monotone and $f(\mathscr{A}_1 \times \cdots \times \mathscr{A}_n) = \mathscr{C}_k$, it inherits for $\mathscr{C}_k$. On the other hand, notice that in $\mathscr{C}_k$ there exists no element from which each element has updistance and downdistance at most $k$, a contradiction. So the monotone onto operations of $\mathscr{C}_k$ are essentially unary, which concludes the proof. □

Let Aut$\mathscr{C}_k$ denote the automorphism group of $\mathscr{C}_k$. We call a tuple

$$\overline{a} = (a_1, \dots, a_n) \in C_k^n$$

a *large range tuple* if none of the $(k+1)$-element subfences of $\mathscr{C}_k$ contain $\{a_1, \dots, a_n\}$. The tuples that are not of large range are called *small range tuples*. For a large range tuple $\overline{a} \in C_k^n$, we define the following relations:

$$R_{\overline{a}} = C_k^n \setminus \{(\sigma(a_1), \dots, \sigma(a_n)) : \sigma \in \text{Aut}\mathscr{C}_k\}.$$

**Lemma 11** *For every large range tuple $\overline{a} \in C_k^n$, $R_{\overline{a}}$ is a critical relation of $\mathscr{C}_k$.*

*Proof* First we prove that the relations in the claim are invariant relations of $\mathscr{C}_k$. For a large range tuple $\overline{a} = (a_1, \ldots, a_n) \in C_k^n$ the relation $R_{\overline{a}}$ has exactly $2k$ missing tuples from $C_k^n$, each of which can be mapped to the other by an automorphism of $C_k$. So to get a contradiction assume, that $\overline{a} = f(\overline{b}, \overline{c}, \ldots)$ for some monotone operation $f$ and tuples $\overline{b}, \overline{c}, \ldots \in R_{\overline{a}}$. However, $f$ must be surjective and, by the preceding theorem, we know that each surjective operation of a crown depends only on a single variable, so, say, $\overline{a} = \sigma(\overline{b})$ for some automorphism $\sigma$ of $\mathscr{C}_k$. This contradicts the fact that $\overline{a} \notin R_{\overline{a}}$ and $\overline{b} \in R_{\overline{a}}$.

Notice that for each large range tuple $\overline{a} = (a_1, \ldots, a_n) \in C_k^n$, $R_{\overline{a}}$ is a coatom in the lattice of the $n$-ary invariant relations of $\mathscr{C}_k$. Hence it is meet-irreducible. So by Lemma 1, $R_{\overline{a}}$ is a critical relation or a direct product of a critical relation and a power of $C_k$. We argue that the latter possibility does not happen. Suppose that it does. Then $R_{\overline{a}}$ is of the form $R \times C_k^l$ where $R$ is an $(n-l)$-ary relation. From the definition of $R_{\overline{a}}$, since $\overline{a}$ has large range, it follows that $|R_{\overline{a}}| = (2k)^n - 2k$. On the other hand, the projection of $(\sigma(a_1), \ldots, \sigma(a_n))$ onto the coordinates of $R$ is outside of $R$ for all $\sigma \in \mathrm{Aut}\mathscr{C}_k$. This yields at least $k(2k)^l$ elements outside of $R_{\overline{a}}$, as every element of $\mathscr{C}_k$ is in a $k$-element orbit of $\mathrm{Aut}\mathscr{C}_k$. So $|R_{\overline{a}}| \leq (2k)^n - k(2k)^l$, a contradiction. Thus, for any large range tuple $\overline{a} \in C_k^n$, $R_{\overline{a}}$ is a critical relation of $\mathscr{C}_k$. □

We remark that it is also of interest to describe a poset that defines the relation $R_{\overline{a}}$ on suitable sequence of elements for every large range tuple $\overline{a} \in C_k^n$. This would yield an alternate proof of the preceding lemma, not using the Demetrovics-Rónyai result. We also note that the preceding lemma easily implies that the Słupecki relation $S_{2k}$ is in the relational clone of $\mathscr{C}_k$, as $S_{2k}$ is the intersection of the $2k$-ary relations $R_{\overline{a}}$ where $\overline{a}$ runs through the $2k$-tuples with pairwise different coordinates. The latter fact was proved by Demetrovics and Rónyai in [3], by exhibiting a poset that defines the Słupecki relation.

For $n \geq 2$, a pair $1 \leq i, j \leq n$ of distinct indices, and a binary relation $S$, we define the $n$-ary relation

$$S^{ij} = \{(a_1, \ldots, a_n) \in C_k^n : (a_i, a_j) \in S\}.$$

In later applications, the arity of the relation $S^{ij}$ will be clear from the context. For $n \geq 2$, a pair $1 \leq i, j \leq n$ of distinct indices, and an $n$-ary relation $T$ let $\pi_{ij}(T)$ denote the projection of $T$ to the coordinates $i$ and $j$. Finally, let $T_{ij}$ denote the $n$-ary relation $= (\pi_{ij}(T))^{ij}$.

**Theorem 12** *Let $L$ be the set of large range tuples in $C_k^n$. For any $n$-ary relation $T$ in the relational clone of $\mathscr{C}_k$ we have that*

$$T = (\cap_{\overline{a} \in L \setminus T} R_{\overline{a}}) \cap (\cap_{1 \leq i < j \leq n} T_{ij}).$$

*Proof* Observe that the right-hand side is an intersection, and, clearly, all the meetands contain the left-hand side. Therefore all we need to prove is that for an arbitrary tuple $b \notin T$, there is a meetand of the right-hand side not containing $b$. For a $b = \overline{a}$ with large range, $R_{\overline{a}}$ suffices. Consequently, we only need to deal with a small range $b$. For a contradiction, let us suppose that $b \notin T$ is contained in all of the meetands of the right-hand side. In this case, we have tuples $b_{ij} \in T$ for all $1 \leq i < j \leq n$ such that $\pi_{ij}(b_{ij}) = \pi_{ij}(b)$. For $b$ has a small range, it is contained in a $(k+1)$-element subfence $\mathscr{F}$ of $\mathscr{C}_k$. As we mentioned at the beginning of this section, there is a retraction $r$ such that $r(C_k) = F$. Applying $r$ to the $b_{ij}$ componentwise, we obtain the tuples $c_{ij}$ in $T$. Since for all $1 \leq i < j \leq n$, $\pi_{ij}(b_{ij}) = \pi_{ij}(c_{ij})$, the $c_{ij}$ interpolate $b$ on every two coordinates. Let $m$ be a majority operation of $\mathscr{F}$. By the Baker-Pixley argument in [1], $b$ is generated from the $c_{ij}$, with the help of the operation $m(r(x), r(y), r(z))$. Thus, $c_{ij} \in T$, $1 \leq i < j \leq n$, yields $b \in T$, a contradiction. □

The following is a straightforward consequence of Theorem 8, Lemma 11 and the preceding theorem.

**Corollary 13** *The critical relations of $\mathscr{C}_k$ are the unary $\emptyset$ relation, the binary relations $U_l$ and $D_l$ for all $l \leq k$, and the relations $R_{\overline{a}}$ for all large range tuples $\overline{a} \in \mathscr{C}_k^n$.*

With little effort, we get the meet-irreducible elements in the lattice of the $n$-ary invariant relations of $\mathscr{C}_k$.

**Corollary 14** *The following is a complete list of the meet-irreducible elements in the lattice* $\mathrm{Inv}^n(\mathrm{Clo}(\mathscr{C}_k))$*:*

1. *$\emptyset$,*
2. *$S^{ij}$ where $S = U_l$ or $S = D_l$ for any $i \neq j$, $i, j \leq n$ and $l \leq k$,*
3. *$R_{\overline{a}}$ for all large range tuples $\overline{a} \in C_k^n$.*

*Proof* By Theorem 12, every meet-irreducible element in $\mathrm{Inv}^n(\mathrm{Clo}(\mathscr{C}_k))$ is of the form as claimed. We only have to prove that $S^{ij}$ is meet-irreducible where $S = U_l$ or $S = D_l$ for $l \leq k$, since the other type relations in the claim are critical by the preceding corollary, and hence they are meet-irreducible. Let $C$ be the intersection of all the relations that are of the form as in the claim and properly contain $S^{ij}$. We shall prove that $C \setminus S^{ij}$ is non-empty. First, observe that if $S^{ij} \subseteq S^{pq}$, then $\{i, j\} = \{p, q\}$. By Theorem 8, there is pair $(a, b)$ in the intersection of the binary invariant relations properly containing $S$ such that $(a, b) \notin S$. Let $c$ be an $n$-tuple whose $i$-th coordinate is $a$ and whose other coordinates are $b$. Then $c$ is a small range tuple, so it is contained in $C \setminus S^{ij}$ . □

## 5 The Subpower Membership Problem for Crowns

In this short section, we prove an easy but interesting corollary that immediately yields a polynomial-time algorithm to decide whether a partial map $f : \mathscr{C}_k^n \rightharpoonup \mathscr{C}_k$ is extendible. So **SMP**($\mathscr{C}_k$) is decidable in polynomial time for any $k$. This can be proved by a direct use of Theorem 10, but here, we give a proof based on the description of the critical relations of $\mathscr{C}_k$.

Let $f : \mathscr{C} \rightharpoonup \mathscr{C}_k$ be a partial map with Dom($f$)= $\{a^1, \ldots, a^l\}$. Then $f$ is called *large range* if the tuple $(f(a^1), \ldots, f(a^l))$ is of large range. We say that $f$ *preserves the up (down) distance* if for any $a, b \in \mathrm{Dom}(f)$

$$\uparrow (f(a), f(b)) \leq \uparrow (a, b) \qquad (\downarrow (f(a), f(b)) \leq \downarrow (a, b)).$$

**Corollary 15** *Let $f : \mathscr{C} \rightharpoonup \mathscr{C}_k$ be a partial map. Then $f$ extends to $\mathscr{C}$ as a fully defined monotone map if and only if there is an $i$ such that*

$$\{(h_i, f(h)) : h = (h_1, \ldots, h_n) \in \mathrm{Dom}(f)\} \subseteq \{(c, \sigma(c)) : c \in C_k\} \tag{5}$$

*for some $\sigma \in Aut\mathscr{C}_k$, or $f$ has a small range and preserves the up and down distances.*

*Proof* We know by Theorem 5 that $f$ is extendible if and only if it preserves all critical relations of $\mathscr{C}_k$. We assume that Dom($f$) is an $l$-element set.

First, suppose that $f$ has large range. Let $\underline{f}$ be the $l$-tuple determined by the values of $f$ on a list of the elements of Dom($f$). Clearly, $\underline{f}$ is a large range tuple. By Lemma 11, the relation $R_{\underline{f}}$ is a critical relation of $\mathscr{C}_k$. Observe that $f$ preserves $R_{\underline{f}}$ if and only if $f$ is extendible. Indeed, if $f$ preserves $R_{\underline{f}}$, then there must be an $i$ such that Eq. 5 holds for some $\sigma \in \mathrm{Aut}\mathscr{C}_k$, that is, $f = \sigma \circ \pi_i|_{\mathrm{Dom}(f)}$, and so $\sigma \circ \pi_i$ is a monoton extension of $f$ to $\mathscr{C}$. Thus, if $f$ has large range, then $f$ is extendible if and only if there is an $i$ such that Eq. 5 holds for some $\sigma \in \mathrm{Aut}\mathscr{C}_k$.

Let us suppose now that $f$ has small range. Then $f$ vacuously preserves all critical relations of the form $R_{\overline{a}}$ for all large range tuples $\overline{a} \in \mathscr{C}$. Then, by Corollary 13, $f$ extends to $\mathscr{C}$ if and only if $f$ preserves the binary critical relations of $\mathscr{C}_k$, which means that $f$ preserves the up and down distances. □

As for any $a, b \in C_k^n$ where $a = (a_1, \ldots, a_n)$ and $b = (b_1, \ldots, b_n)$

$$\uparrow (a, b) = \max_{1 \le i \le n} \uparrow (a_i, b_i) \text{ and } \downarrow (a, b) = \max_{1 \le i \le n} \downarrow (a_i, b_i)$$

in $\mathscr{C}$, all conditions of the corollary check in polynomial time. Thus **SMP**($\mathscr{C}_k$) is decidable in polynomial time for any $k$. This implies that **RExt**($\mathscr{C}_k$) is also decidable in polynomial time. On the other hand, as we mentioned in the introduction, **Ext**($\mathscr{C}_k$) is **NP**-complete.

**Funding** Open access funding provided by University of Szeged.

**Open Access** This article is licensed under a Creative Commons Attribution 4.0 International License, which permits use, sharing, adaptation, distribution and reproduction in any medium or format, as long as you give appropriate credit to the original author(s) and the source, provide a link to the Creative Commons licence, and indicate if changes were made. The images or other third party material in this article are included in the article's Creative Commons licence, unless indicated otherwise in a credit line to the material. If material is not included in the article's Creative Commons licence and your intended use is not permitted by statutory regulation or exceeds the permitted use, you will need to obtain permission directly from the copyright holder. To view a copy of this licence, visit http://creativecommons.org/licenses/by/4.0/.

## Data Availability

Data sharing not applicable to this article as no datasets were generated or analysed during the current study.
